# Assessing data analysis techniques in a high-throughput meiosis-like induction detection system

**DOI:** 10.1186/s13007-023-01132-9

**Published:** 2024-01-12

**Authors:** Tanner M. Cook, Eva Biswas, Somak Dutta, Siddique I. Aboobucker, Sara Hazinia, Thomas Lübberstedt

**Affiliations:** 1 Department of Agronomy, Iowa State University, Ames, IA USA; 2 Department of Statistics, Iowa State University, Ames, IA USA

**Keywords:** In vitro biology, Meiosis induction, Single-cell analysis, Plant breeding and biotechnology

## Abstract

**Background:**

Strategies to understand meiotic processes have relied on cytogenetic and mutant analysis. However, thus far in vitro meiosis induction is a bottleneck to laboratory-based plant breeding as factor(s) that switch cells in crops species from mitotic to meiotic divisions are unknown. A high-throughput system that allows researchers to screen multiple candidates for their meiotic induction role using low-cost microfluidic devices has the potential to facilitate the identification of factors with the ability to induce haploid cells that have undergone recombination (artificial gametes) in cell cultures.

**Results:**

A data analysis pipeline and a detailed protocol are presented to screen for plant meiosis induction factors in a quantifiable and efficient manner. We assessed three data analysis techniques using spiked-in protoplast samples (simulated gametes mixed into somatic protoplast populations) of flow cytometry data. Polygonal gating, which was considered the “gold standard”, was compared to two thresholding methods using open-source analysis software. Both thresholding techniques were able to identify significant differences with low spike-in concentrations while also being comparable to polygonal gating.

**Conclusion:**

Our study provides details to test and analyze candidate meiosis induction factors using available biological resources and open-source programs for thresholding. RFP (PE.CF594.A) and GFP (FITC.A) were the only channels required to make informed decisions on meiosis-like induction and resulted in detection of cell population changes as low as 0.3%, thus enabling this system to be scaled using microfluidic devices at low costs.

**Supplementary Information:**

The online version contains supplementary material available at 10.1186/s13007-023-01132-9.

## Background

Meiosis is a unique cellular process that is thought to have evolved once in eukaryotes [8]. This process or its precursors have been induced in species outside of plants using various factors [6, 7, 9]. Medrano et al., [13] provided evidence for the induction of a germ-cell like phenotype from somatic cells with the ectopic expression of six genes, subsequently, 1% of these cells underwent meiosis. These results suggest that in vitro meiosis induction might be possible in plants as well, while potentially requiring only a limited number of factors. If possible, in vitro meiosis induction would enable plant breeders to maximize the number of generations grown per year by circumventing the need for flowering. Here, we aim to provide a resource to determine key regulators in the switch from mitosis to meiosis in the absence of flowers as a critical step to enable breeding in vitro.

**Fig. 1:**
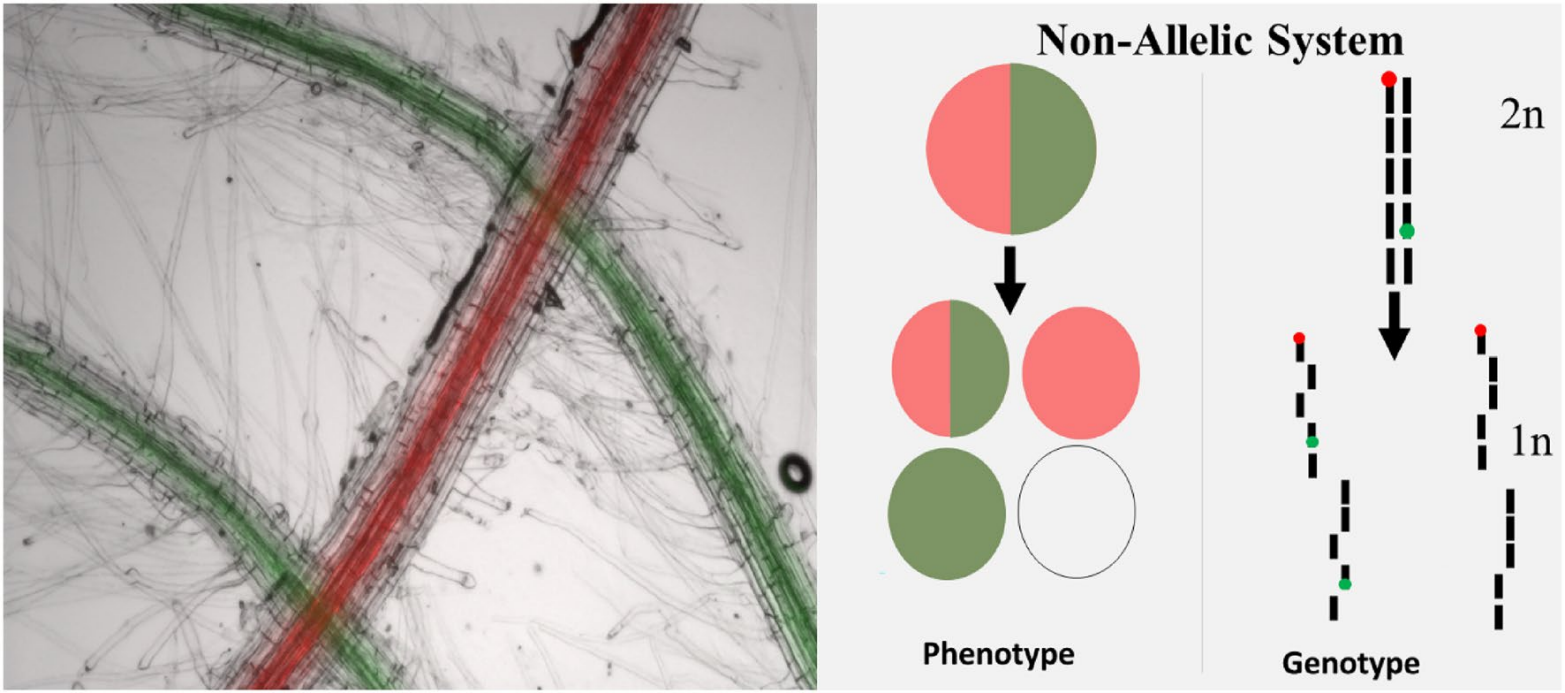
Bi-fluorescent marker system for high-throughput meiosis-like induction detection. RFP and GFP fluorescent Arabidopsis root images (left). Depiction of chromosomal segregation detection using a non-allelic fluorescent system in protoplasts after meiosis-like induction (right). Cells depicted as containing both GFP and RFP are identified with red and green ellipses within the same larger circle, cells that contain only RFP are depicted by a red circle, cells that contain only GFP are depicted by a green circle, and non-fluorescing cells are depicted by a white circle. RFP and GFP genes are represented by small red and green circles, respectively on chromosomes

Single-cell approaches to plant research were established decades ago [4], but recent developments have provided single cells as a robust research tool. Significant advances include single-cell RNA sequencing [12] or pseudotime velocity assessments of plant developmental stages [17]. Protoplasts, microspores, and nuclei have also been isolated and used as a way to study individual cell groups through fluorescence activated cell sorting [1, 2, 18], thus enabling data to be collected at the cell-type level. Recently, we have proposed and built a bi-fluorescent tool to detect meiosis-like induction in high-throughput with protoplasts isolated from callus [5], Cook et al., unpublished, Fig. 1). Potentially low induction rates and a number of potential induction factors require that this system can be scaled to analyze many cells simultaneously. We developed this tool in *Arabidopsis thaliana* due to the vast number of genetic resources and mutants available in this species as well as the short time between generational cycles. Additionally, even though *Arabidopsis* is a dicotyledonous species, cell cycle processes are conserved among plants. Evidence for this is provided in the MiMe mutants, where cells will undergo mitosis instead of meiosis. Mutations in three genes led to this meiotic disruption in both *Arabidopsis* and rice [15].

This methods paper is a detailed compilation of various protocols to enable meiosis induction testing while providing opportunities to adapt our system with the use of microfluidic devices. The objectives of this work are to: (i) provide a detailed procedure for developing a high-throughput system for studying single-cells while also providing a pipeline to convert internationally recognized .fcs formatted files [21], https://isac-net.org/page/Data-Standards) to.csv files for analysis using open-source software. (ii) Critically assess sophisticated polygonal gating techniques and open-source thresholding to (iii) determine the minimal number of fluorescent channels necessary to detect small population shifts in simulated meiosis induction studies (haploid cells among a large population of diploid cells).

## Materials and methods

### Plant materials development protocol

**Fig. 2:**
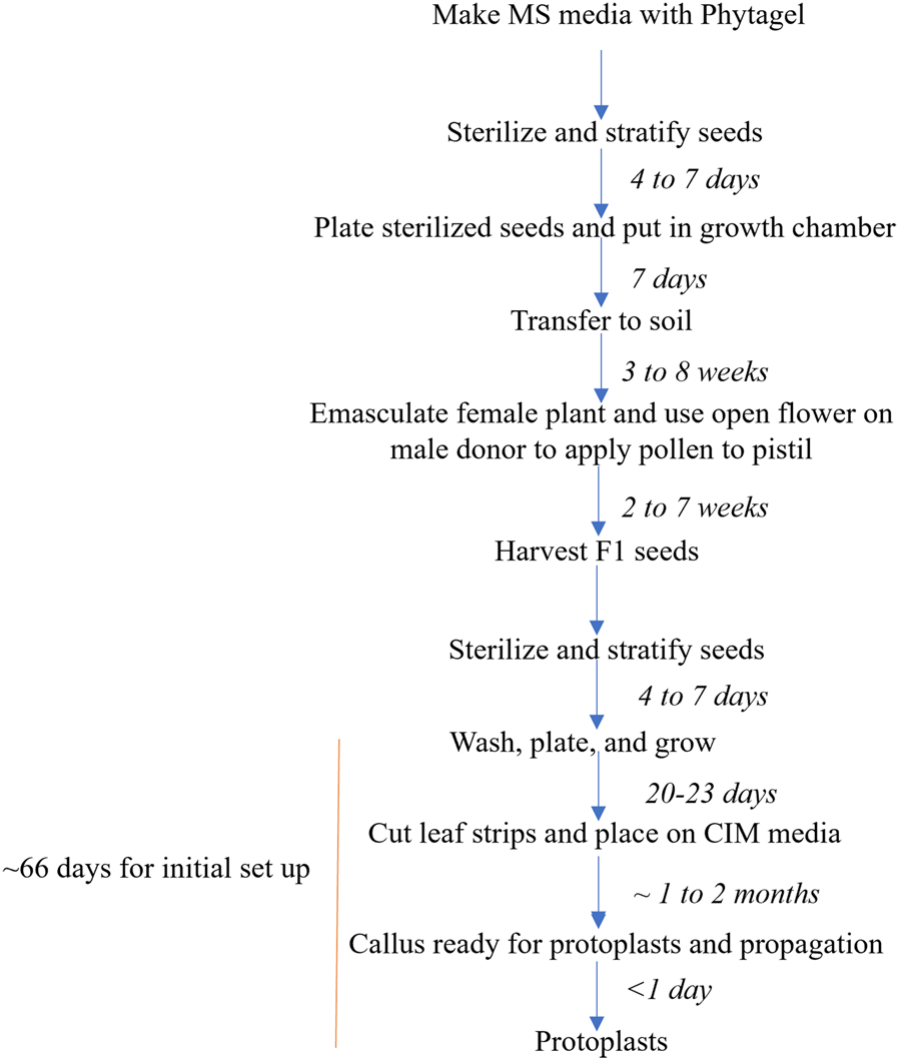
Summary of steps to develop a high-throughput meiosis-like induction analysis system

Two homozygous *Arabidopsis* lines were developed using *Agrobacterium tumefaciens*-mediated transformation, each single-locus insertion lines, one carrying a 35S::eGFP::NosT and the other carrying a 35 s::mRFP::NosT. *A. thaliana* ecotype Col-0 (transgenic and wildtype) seeds were surface sterilized in microcentrifuge tubes for 5-min in 70% ethanol and for 10-min in 1:1 bleach (do not exceed 20-min). The seeds were rinsed 5–7 times under sterile conditions and stratified in 1 mL of sterile water at 4 °C for four to seven days. After stratification, seeds were plated on square plates containing full-strength MS media (Fig. 2; [16]). Four seeds per grid square of diploid plants were sown. Plates containing the seeds are then incubated at 23–24 °C in 16/8 h light/dark cycles. Diploid plants used for crossing can be directly sown on soil and stratified or transferred to soil from MS plates after 7 days and continued to grow under the same lighting and temperature conditions. To develop GFP/RFP F1 plants, we gently emasculated unopened flower buds of plants from the GFP line and applied pollen from plants of the RFP line to the exposed pistil (Fig. 2, [11]). Haploid RFP plants were developed following Ravi and Chan [20], where the RFP line was the male donor. After maturation of the silique, seeds were collected and dried at 37 °C for 12–24 h. The seeds were subsequently stored at 4 °C in a dry tube.

### Callus initiation

**Fig. 3:**
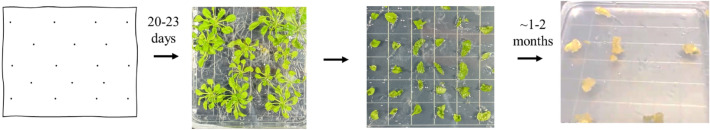
Callus induction media. Schematic for callus initiation steps. Callus image was taken after leaf fragments were grown in the dark on CIM for 27 days. Callus image enhanced for clarity

F1 GFP/RFP seeds and controls (RFP, GFP, and WT genotypes) were planted as described above with approximately 15 to 20 seeds per plate. The seedlings were allowed to grow under light conditions for 20 to 23 days (Figs. 2 and 3). Genotyping the F1 can be done to ensure absence of cross contamination, but we found this to be unnecessary. Under sterile conditions we used forceps and a razor to remove leaf fragments and placed them on callus induction media (Fig. 3: modified from [23]). Plates were covered with parafilm and placed in complete darkness at 23–24 °C. After approximately one to two months, enough callus was present for protoplast isolation. Note—Callus should be sub-cultured every 2 to 4 weeks for the best results.

### Protoplast isolation

For protoplast isolation, we used a protocol modified from Yoo et al. (26, see Recipes). Eight to ten milliliters of sterile enzyme mix containing macerozyme and cellulase was added to a petri dish. Whitish callus pieces were added to the mix and chopped into smaller pieces using a razor blade under sterile conditions. Note—Healthier callus will yield more protoplasts. The mixture was allowed to incubate for approximately 3 h in a laminar flow hood in the dark. After incubation the mixture was swirled to aid in the release of protoplast isolation, W5 was then added to the mixture 1 mL at a time up to 5 mL, making the final volume 13 to 15 mL. The sample was swirled and passed through a 40 µM filter into a clean 50 mL Falcon tube using a cut 1000 µL pipette tip (sterility is not critical at this time). Five more milliliters of W5 were added to the petri dish containing the callus, swirled, and passed again through the 40 µM filter. The resulting mixture should be ~ 20 mL. Note—cover the solution with foil whenever possible to avoid photobleaching. The samples were centrifuged using a swing-bucket centrifuge at 100*g*×5-min using reduced brake and acceleration. Supernatant was removed and 5 mL of W5 added to the Falcon tube containing the cells, then gently resuspended. Cells were transferred into a 15 mL round bottom tube and centrifuged at 100*g*×2-min using moderate brake and acceleration. Again, supernatant was removed and MMG was added to the desired cellular concentration.

### Spike-in testing

To test the system and compare analysis techniques, small percentages of callus-derived protoplasts from the haploid RFP line were added to callus-derived protoplasts from the diploid GFP/RFP genotype, the cells were mixed thoroughly together, and the samples were then run through a flow cytometer for analysis.

### Flow cytometry and data analysis

Freshly isolated protoplasts stored in MMG solution were run through a flow cytometer with 0.2 µm-filtered 1X PBS for instrument sheath fluid. GFP and RFP fluorescence analysis of protoplasts was performed with a factory direct, unmodified BD FACSCanto (BD Biosciences; San Jose, CA) using fluorescent compensation by the Iowa State University Office of Biotechnology Flow Cytometry Facility. Instrument performance was monitored regularly by Flow Cytometry Facility staff using the BD Biosciences Cytometer Set up & Tracking calibration bead system. Instrument calibration and gating for data analyses were verified within each experiment by use of appropriate single-color control samples. The laser used had a wavelength of 488 nm at 20mW with GFP emission filters of 525/50 nm and RFP emission filters of 610/20 nm. Intact protoplasts were identified using a combination of light scatter and autofluorescence properties. BD FACSDiva (v.8.0.1) software was used for general data acquisition and analysis for polygonal gating. For thresholding, FlowCal [3] and R version 4.1.3 [19] with packages ggplot2 version 3.3.6 [24], dplyr version 1.0.8 [25] and ggpubr version 0.4.0 [10] were used for file conversion, thresholding, and visualization.

### Statistical considerations

We explored three methods of classification based on gates. Each classifier categorizes the data into four groups, and generates counts for RFP, WT, GFP + GFP/RFP (combining the counts of GFP and GFP/RFP). However, these counts suffer from misclassification, in particular, the class WT actually contains the cells either RFP or GFP/RFP. So, to accommodate the misclassified cells, we adopt the multinomial model, which is consistent across all three classifiers. Hence, we focus on one classifier, say the polygonal gating, and discuss the modeling technique. Let $\{{y}_{il1},{y}_{il2},{y}_{il3}\}$ be the counts of RFP, GFP + GFP/RFP, and WT obtained from the data sets with ${l}^{th}$ level of spike-in from the ${i}^{th}$ set of experiments. Here for *l* = *1* the data exclusively contains GFP/RFP, hence the counts under the categories RFP (*y*_*i11*_) and WT (*y*_*i13*_) are all misclassifications. Similarly, for *l* = *2*, the cells are all RFP, indicating *y*_*i22*_ and *y*_*i23*_ are the misclassification counts. Let ${q}_{i1}$, ${q}_{i2}$, and ${q}_{i3}$ denote the probability of a cell being classified as RFP, GFP + GFPxRFP, and WT, respectively, when it is actually GFP or GFP/RFP. Similarly, define ${p}_{i1}$, ${p}_{i2}$, and ${p}_{i3}$ be the probability of a cell being classified as RFP, GFP + GFP/RFP, and WT, respectively, if it is actually an RFP. Additionally, let ${s}_{il}>0$ be the true spike-in (given in proportion) of the ${l}^{th}\left(>2\right)$ data set from the same experiments group $i$. Then for the *i*^th^ experiment and the *l*^th^ spike-in data, we calculate the probabilities,

$$P\left(a \,cell \,identified \,as \,RFP\right)={p}_{i1}{s}_{il}+{q}_{i1}\left(1-{s}_{il}\right), P\left(a \,cell \,identified \,as \,GFP+GFPxRFP\right)={p}_{i2}{s}_{il}+{q}_{i2}\left(1-{s}_{il}\right),$$

and

$$P\left(a\, cell \,identified \,as \,WT\right)={p}_{i3}{s}_{il}+{q}_{i3}\left(1-{s}_{il}\right).$$

These *misclassification*-*adjusted* probabilities are shown in Table 1.

**Table 1:** Cell classification probabilities

Spike-ins proportion	Prob of RFP	Prob of GFP + GFP/RFP	Prob of wild type
0 (*l* = *1*)	${q}_{i1}$	${q}_{i2}$	${q}_{i3}$
1 (*l* = *2*)	${p}_{i1}$	${p}_{i2}$	${p}_{i3}$
${s}_{il}>0$(*l* > *2*)	${p}_{i1}{s}_{il}+{q}_{i1}\left(1-{s}_{il}\right)$	${p}_{i2}{s}_{il}+{q}_{i2}\left(1-{s}_{il}\right)$	${p}_{i3}{s}_{il}+{q}_{i3}\left(1-{s}_{il}\right)$

The probability of a cell being classified as RFP or GFP + GFP/RFP or wild type are presented with the rows denoting different values of spike-ins ${s}_{il}, l=\mathrm{1,2},\dots ,{L}_{i}$

Combining all the data sets from the ${i}^{th}$ experiment, the log-likelihood is written as:

$$l\left({{\varvec{q}}}_{{\varvec{i}}.},{{\varvec{p}}}_{{\varvec{i}}.},{{\varvec{s}}}_{{\varvec{i}}.}\right)={\sum }_{l=1}^{{L}_{i}}{\sum }_{j=1}^{3}{y}_{ilj}log\left({p}_{ij}{s}_{il}+{q}_{ij}\left(1-{s}_{il}\right)\right).$$

To estimate the parameters we maximize the log-likelihood. Finally, ${\widehat{s}}_{il}:l=3,\dots ,{L}_{i}$ provide the set of estimated spike-ins for the ${i}^{th}$ experiment.

## Results

**Fig. 4:**
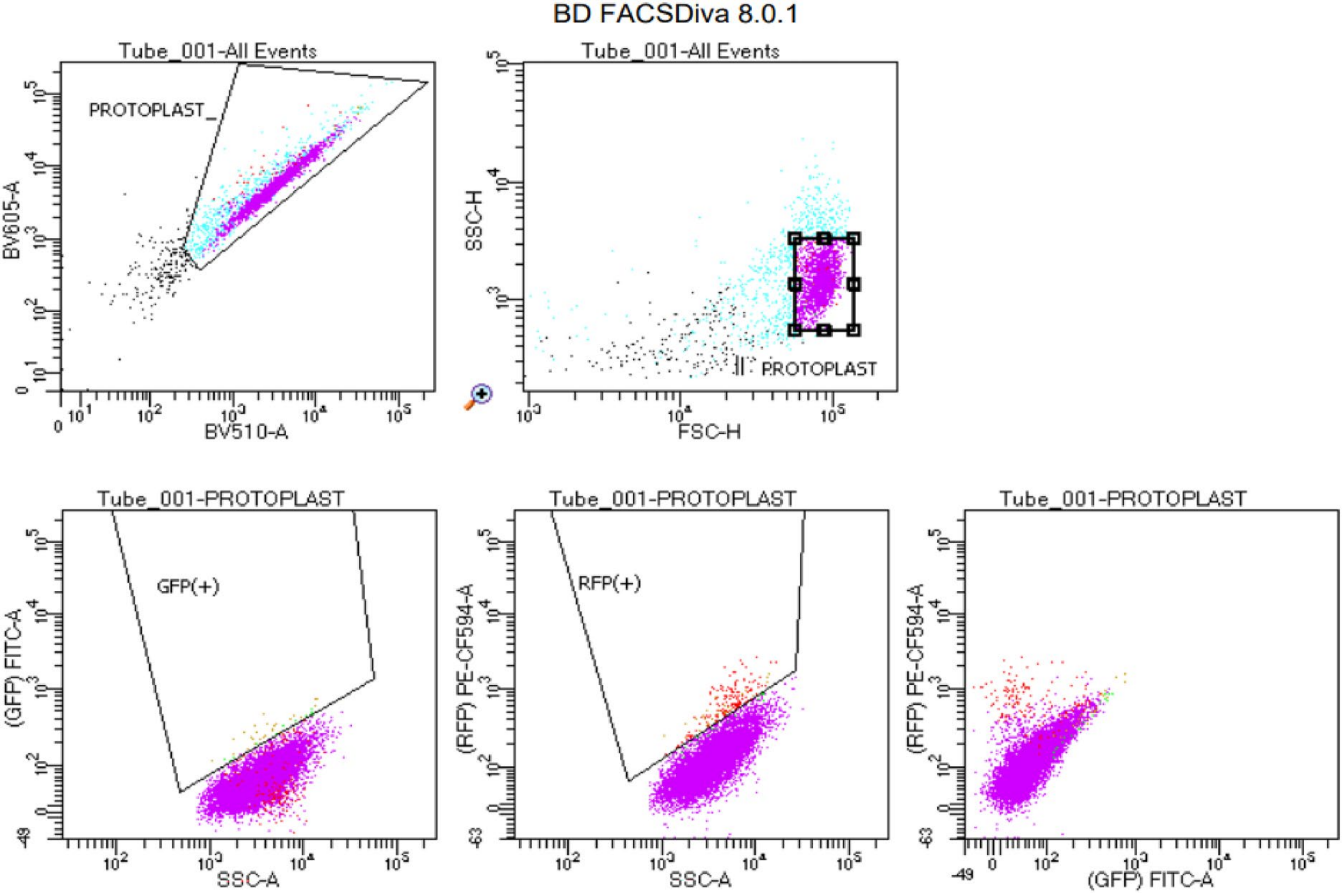
Example of cell classifications with the BD FACSDiva (v.8.0.1) software for polygonal gating. Values are the basis for thresholding quality values

In order to determine the number of necessary fluorescent channels to detect simulated gametes in large diploid cell populations and whether open-source analyses could be used, polygonal gating and thresholding (quality, RFP, and GFP; or only RFP and GFP) were compared. Figure 4 is an example of the polygonal gating technique from the BD FACSDiva (v.8.0.1) software where raw data was initially classified as protoplasts based on autofluorescence properties and then further refined using forward and side scatter to determine physical characteristics consistent with protoplasts. Following refinement, cells were then defined in regard to their RFP and/or GFP fluorescence. For thresholding, maximum and minimum values of multiple channels were used with open-source software to define quality protoplasts (if quality thresholds were used) and red and green fluorescing cells (Table 2). The values from Fig. 4 were estimated to develop the maximum and minimum mean fluorescence intensity values for quality thresholding (quality, RFP, and GFP) (Table 2). Additionally, RFP and GFP were defined using only FITC.A and PE.CF594.A with the thresholding analyses as compared to polygonal gating, which used side scatter and FITC.A or PE.CF594.A to define the ranges of RFP and GFP for each cell population.

**Table 2:** Thresholding values based on mean fluorescence intensity values

Filter name	Filter	Thresholding for only RFP and GFP			Thresholding for quality, RFP, and GFP		
		RFP Gates	GFP Gates	Both Gates	RFP Gates	GFP Gates	Both Gates
FSC.H	Forward scatter height	No threshold applied	No threshold applied	No threshold applied	> 55,000	> 55,000	> 55,000
FSC.H	Forward scatter area	No threshold applied	No threshold applied	No threshold applied	< 200,000	< 200,000	< 200,000
SSC.H	Side scatter height	No threshold applied	No threshold applied	No threshold applied	> 450	> 450	> 450
SSC.H	Side scatter height	No threshold applied	No threshold applied	No threshold applied	< 3500	< 3500	< 3500
	**Excitation/emission**						
BV605.A	407/602*	No threshold applied	No threshold applied	No threshold applied	> 800	> 800	> 800
BV510.A	405/510*	No threshold applied	No threshold applied	No threshold applied	> 800	> 800	> 800
FITC.A	494/520*	< 560	> 560	> 560	< 560	> 560	> 560
FITC.A	494/520*	N/A	< 10,000	< 10,000	N/A	< 10,000	< 10,000
PE.CF594.A	496,566/612*	> 1000	< 1000	> 1000	> 1000	< 1000	> 1000
PE.CF594.A	496,566/612*	< 10,000	N/A	< 10,000	< 10,000	N/A	< 10,000

Table 2 provides the minimum and maximum mean fluorescence intensity values used for both thresholding techniques. Quality thresholding used FSC.H, SSC.H, BV605.A, and BV510.A, while FITC.A and PE.CF594.A were used to define RFP and GFP. Quality thresholds were applied to only one of the thresholding methods (quality, RFP, and GFP), while GFP and RFP thresholds were applied to both of the thresholding methods.

**Fig. 5:**
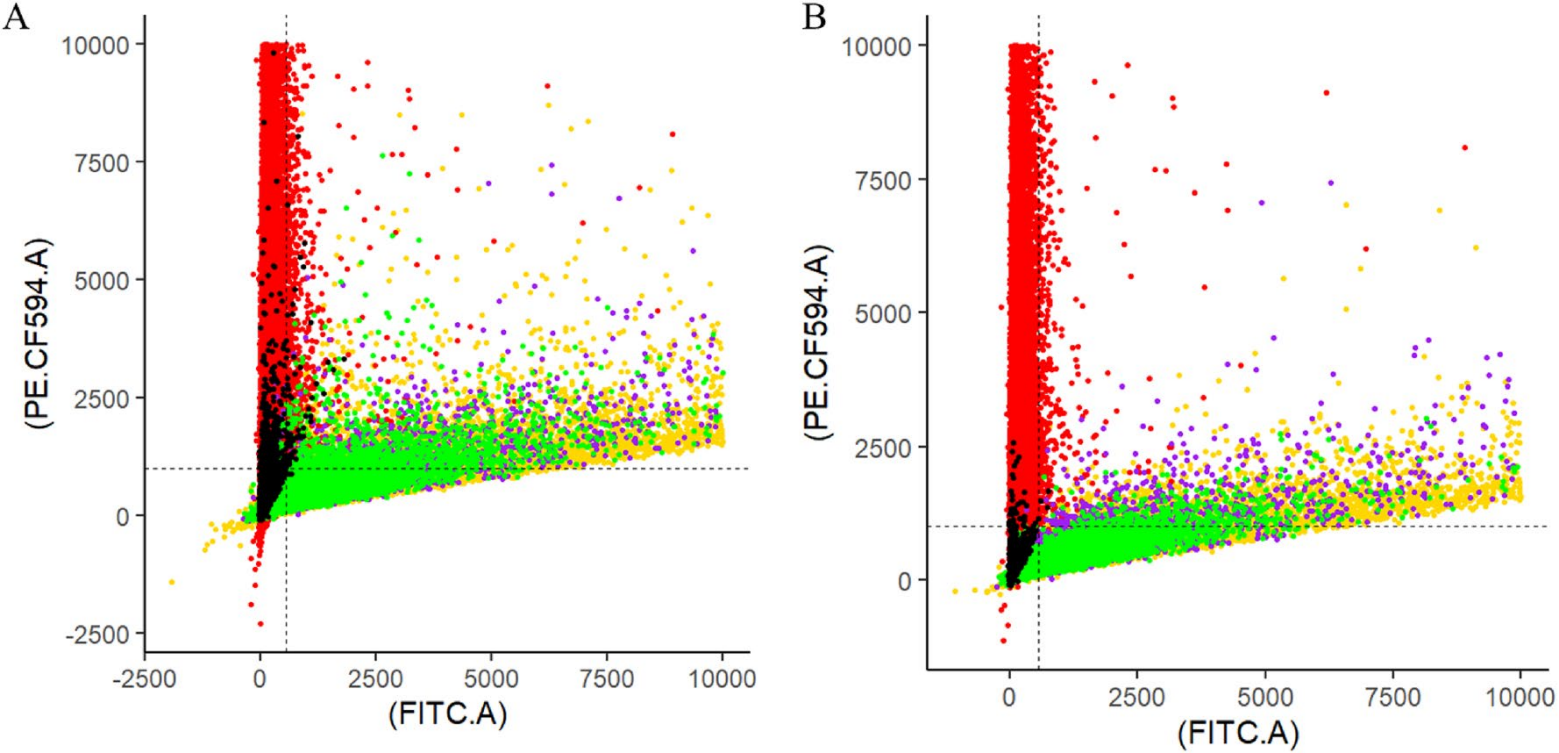
Thresholding determination. Thresholding for only RFP and GFP (left). Thresholding for quality, RFP, and GFP (right). Colors represent all cells from an individual sample or a collation of multiple samples without RFP and GFP thresholds (10,000 maximum was applied). Red represents callus protoplasts from the RFP line collated from 05-02-2022 and 06-24-2022. Green represents callus protoplasts from the GFP line from 05-02-2022 and 06-24-2022. Gold represents callus protoplasts from GFP/RFP (Plant #10) genotype collated from 05-02-2022 and 06-24-2022. Purple represents callus protoplasts from GFP/RFP (Plant#12) genotype from 01-13-2022. Black represents callus protoplasts from the WT line from 01-13-2022. Black dotted lines represent the thresholds for RFP (PE.CF594.A; Horizontal) and the thresholds for GFP (FITC.A;Vertical) used for analysis

To compare the two thresholding methods, we assessed where cell populations were positioned in regard to the RFP and GFP thresholds. Figure 5 provides the visualization of overlapping, non-classified cell populations from different genotypes (RFP, GFP, GFP/RFP, and WT) with the FITC.A and PE.CF594.A thresholds marked. This provides evidence for the effectiveness of the RFP and GFP thresholding values, which could be even further fine-tuned using our analysis pipeline. Cells from the RFP plant line are well accounted for with the FITC.A and PE.CF594.A thresholds regardless of quality thresholding. Cells from WT and GFP plant lines are better accounted for when quality thresholding is used. Further, cells from the GFP and GFP/RFP plant genotypes were not well separated in either thresholding analysis.

**Fig. 6:**
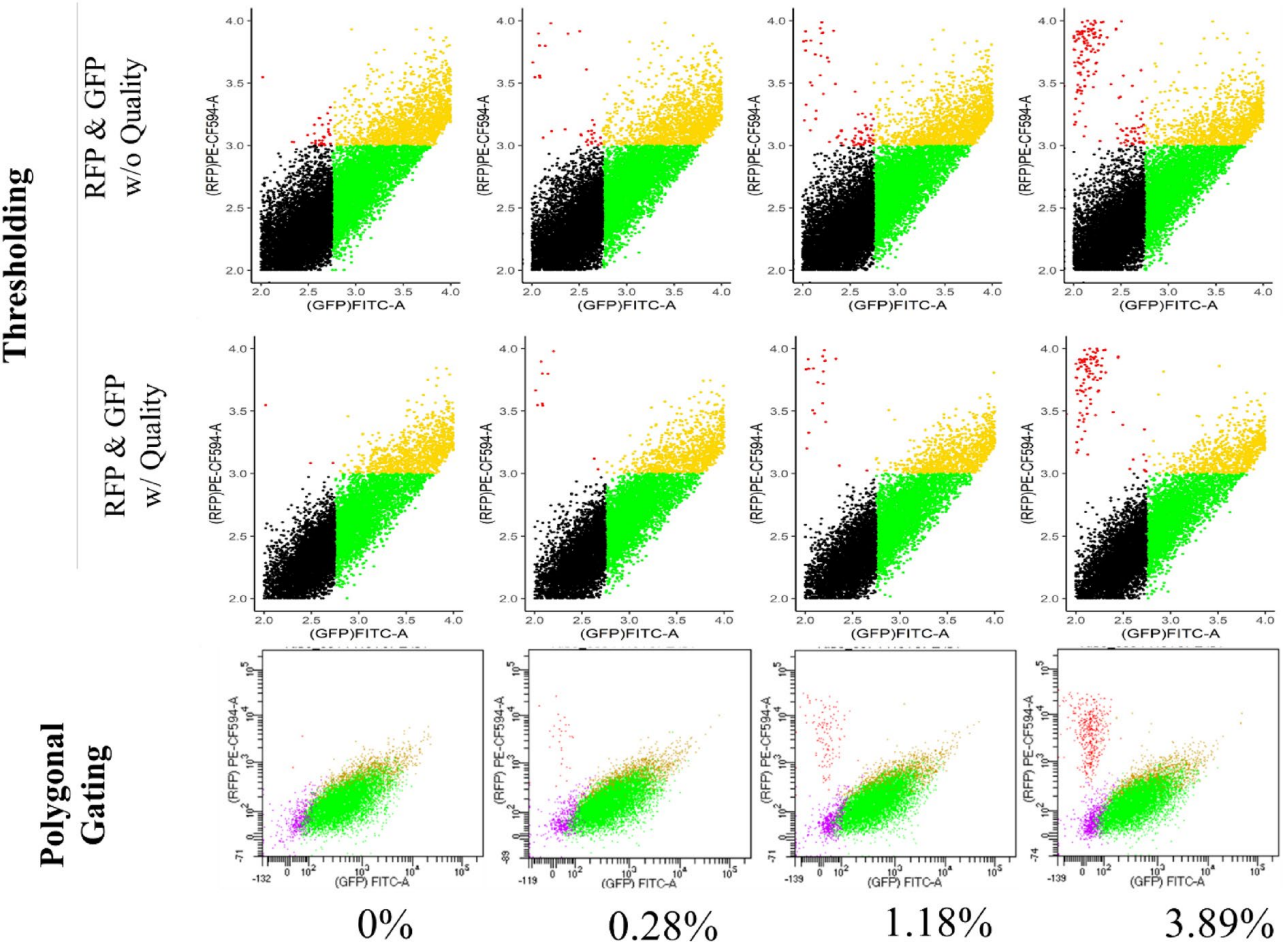
Analysis of fluorescent protoplasts. Analysis of flow cytometry data as individual cells are graphed against the log of RFP (PE.CF594A) and log of GFP (FITC.A). Thresholding for only RFP and GFP (Top row), thresholding for quality, RFP, and GFP (Middle row), polygonal gating (Bottom row). Left to right in each row: 0.0% RFP spike-ins, 0.28% RFP spike-in, 1.18% RFP spike-in, 3.89% RFP spike-in. Black and purple points represent cells classified as WT, green points are cells classified as GFP, gold points represent cells that are classified as GFP/RFP, and red points represent those classified as RFP. Spike-in percentages were determined by polygonal gating. Axis limits were between 2 and 4 for both thresholding techniques. Histograms for each scatterplot are shown in the Additional file 1

We looked at populations of cells from the diploid GFP/RFP genotype that were spiked with cells from a haploid RFP line to compare polygonal gating and thresholding (Fig. 6). Wald tests were used to test the detectability of RFP cells using each gating analysis method (Table 3). For this, each GFP/RFP control was analyzed by the respective analysis (i.e. polygonal gating or thresholding) and compared to the spike-in using that same analysis (Table 3).

**Table 3:** RFP identification

Date	Thresholding				Polygonal gating	
	Percent red cells detected with quality thresholding + RFP and GFP	P-value from Wald test (compared to control)	Percent red cells detected with only GFP and RFP	P-value from Wald Test (compared to control)	Percent red cells detected with Polygonal gating	P-value from Wald Test (compared to control)
220502	1.78%	< 0.00001	1.82%	< 0.00001	2.2%	< 0.00001
220502	3.18%	< 0.00001	3.05%	< 0.00001	3.69%	< 0.00001
220502	33.71%	< 0.00001	30.19%	< 0.00001	38.64%	< 0.00001
220624	0.22%	0.00009	0.16%	0.01332	0.28%	< 0.00001
220624	0.76%	< 0.00001	0.89%	< 0.00001	1.18%	< 0.00001
220624	3.17%	< 0.00001	2.8%	< 0.00001	3.89%	< 0.00001

Comparisons of RFP identification from thresholding for quality, RFP, and GFP; thresholding for only RFP and GFP; and polygonal gating

As can be seen in Fig. 6, all three analysis types had population separations of RFP classified cells from those classified as GFP and GFP/RFP. When quality thresholding was not applied, there appeared to be two populations of RFP classified cells, one that was distinct and another that was close to the GFP and GFP/RFP classified cell populations. Within all three analyses, GFP was not a distinct population apart from GFP/RFP classified cells. Further, the line separating cells classified as WT and those classified as GFP was not as defined either.

**Fig. 7:**
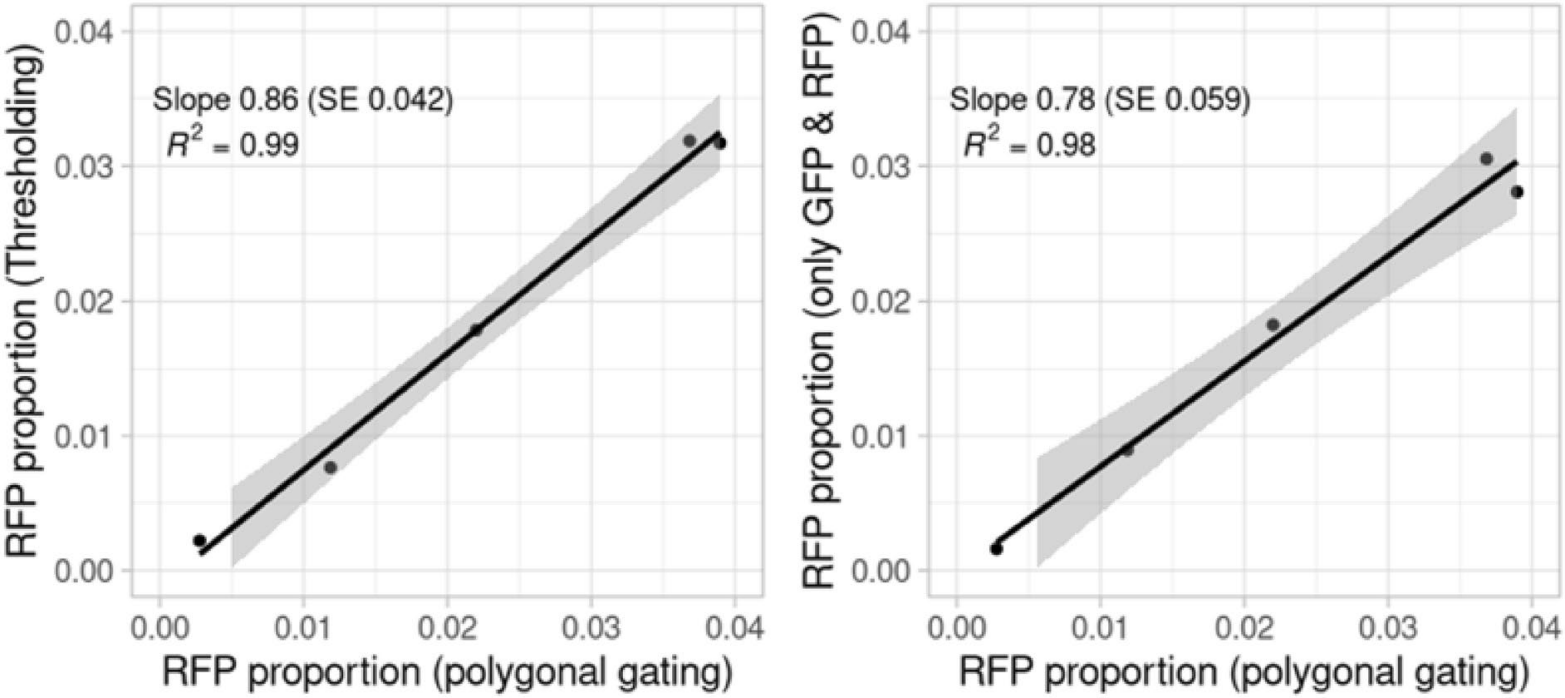
Regression data. Regressions of detected RFP proportions from thresholding for quality, RFP, and GFP (left) and thresholding for only GFP and RFP (right) on the detected RFP cell proportion obtained from polygonal gating

Since cells from the RFP line were spiked into cells from the GFP/RFP genotype to simulate meiosis induction, we determined the amount of detected RFP cells as a proportion of the total number of cells for comparison (Table 3). Data from each gating technique was analyzed by a multinomial model (see “Statistical considerations”) to estimate the percentage of RFP cells present in the spike-ins after accounting for misclassifications. The p-values from Wald tests provide strong evidence that detected spike-ins as low as 0.3% are observable by all gating methods. For the lowest spike-in sample, thresholding for only RFP and GFP without quality thresholds resulted in a detection of 0.16% RFP cells, and when compared to the GFP/RFP samples without spike-ins, a p-value of 0.013 was obtained (Table 3). However, polygonal gating provided larger estimates of detected RFP cell percentage rates the other gating methods. Detected RFP cell proportions from thresholding for quality, RFP, and GFP and thresholding for only GFP and RFP were regressed on the detected RFP cell proportion obtained from polygonal gating after removing an influential point, and the regression coefficient was used to measure the overall discrepancy in detection rates (Fig. 7). These simple linear regression analyses suggest that, with polygonal gating as the baseline, the estimated recovery rates of thresholding for quality, RFP, and GFP and thresholding for only RFP and GFP are 86% (SE 4.2%) and 78% (SE 5.9%), respectively.

## Discussion

Determining the data points necessary to make informed decisions on detecting meiosis-like induction using our outlined system provides evidence that RFP (PE.CF594.A) and GFP (FITC.A) fluorescence are the minimum in which our system can be assessed. By reducing the need for large amounts of collected data to make decisions, this system can easily be scaled with the use of low-cost microfluidic devices, which are becoming increasingly well-researched [14]. With microfluidics, we envision researchers taking callus cultures that have been treated with candidate meiosis induction factors and isolating protoplasts. These cells can then be passed through a microfluidic device that is equipped with an excitation laser and only two detectors, RFP and GFP, and analyzed for a shift in fluorescent cell populations. Without the need for multiple detectors, microfluidic devices could replace flow cytometers for meiosis induction testing as they can be built with minimal hardware and still provide robust data for meiosis induction evaluation while also opening the door to parallel devices being run.

Open-source data analysis packages, such as the ones used in this study, enable researchers with minimal resources to fully take advantage of the tools presented. Further, the pipeline provided allows researchers to convert .fcs files to .csv files so they can fine-tune and assess their analysis without the need for a skilled flow cytometry expert once data is collected. Having the ability to make micro-adjustments to thresholds without the need for protoplast quality gating provides a user-friendly and efficient analytical system that can easily be scaled with a minimal background in command-line coding. As a note, polygonal gating by a professional provides important detection that was more refined in the analyses (Fig. 4). However, significant differences in RFP population distributions were still found in all of the spiked-in samples analyzed in this study, regardless of the analysis technique.

The advantage of the method presented in this article is that meiosis induction factors can be tested in around 66 days in a lab setting with equipment that can be found in any plant research laboratory. Further, once callus is established, good practices can be used to keep callus cultures for several months, thus providing a constant source of materials for meiosis induction factor testing. This system also eliminates the need for dyes and other reagents as fluorescent markers are already present in the genome. One of the disadvantages of our system, however, is that by using *Arabidopsis* we will most likely need to adapt the conditions or factors that induce meiosis in vitro when testing in crop species. Additionally, requiring tissue to undergo callus formation is time-consuming and not currently possible in some species. Because of this, we do not necessarily suggest that this is the best system for meiosis-like induction to be used in in vitro breeding programs, but should instead be used as a tool to identify induction factors.

This methods paper builds on the work we have been conducting in order to develop tools for plant researchers. In summary, we have (i) provided a set of tools to implement a high-throughput, single-cell meiosis-like induction detection system that can be run through a flow cytometer and analyzed with minimal computing and software resources. (ii) We assessed various gating and thresholding techniques to (iii) make decisions on meiosis-like induction data while only requiring RFP and GFP fluorescence detection. By establishing callus, treating the cells, and isolating protoplasts for analysis, a streamlined approach to determine the underpinnings of meiosis induction in plants can be scaled to testing multiple candidate factors.

## Step-by-step implementation

When first setting up such a system, it is important to have the appropriate controls so the data collection and analysis are successful. A very important note—The threshold values will need to be determined for each flow cytometer and settings need to be consistent. We recommend having WT, GFP, RFP, and GFP/RFP controls when first determining appropriate thresholds and flow cytometer settings, but after initial setup, GFP/RFP cells are sufficient as controls with occasional RFP and GFP checks. Below is a step-by-step procedure including materials and reagents, equipment (Table 4), recipes, and procedures to recreate the system we have outlined in this methods paper.

**Table 4:** Equipment

Sterile Laminar Flow Hood	
Autoclave	
Incubator for 23-24 ºF	
Flow Cytometry facility	
1 mL Pipette tips	
Square culture plates	
Growth Chamber	
Fine tipped forceps	
Magnifying glasses	
Thermocycler	
Dry bath	
50 mL Falcon tubes	
Magenta Boxes	

Materials and reagents:

1. Arabidopsis lines
  1. GFP 1–3(ABRC Accession No.: CS73496)
  2. RFP 2–3 (ABRC Accession No.: CS73495).
2. Murashige and Skoog Solid Media [16]
  i) MS Salts (Caisson Laboratories Inc Ref #: MSP01 or Sigma-Aldrich see below)
  ii) MS Vitamin Mix (1000x) (Caisson Laboratories Inc Ref#: MVL01)( We have used Sigma-Aldrich Reference #: M5519 as well, which contains salts and vitamins)
  iii) Sucrose (Store bought)
  iv) Phytagel (Sigma Aldrich Ref#: P8169)
  v) MgSO_4_*7H_2_O
  vi) KOH.
3. Seed sterilization
  i) Concentrated bleach (6 or 8% sodium hypochlorite)
  ii) 100% Ethanol
  iii) Sterile double deionized water.
4. Callus induction medium [23]
  i) 2, 4-dichlorophenoxyacetic acid (2, 4-D) (Sigma-Aldrich Ref #: D70724)
  ii) 6-benzylaminopurine (6-BA) (Acros Organics Ref #: 226410050)
  iii) Parafilm
  iv) Sterilization Filters
  v) MS media w/o Phytagel and MgSO_4_*7H_2_O (see above)
  vi) Bacto Agar (BD Ref #: 214010).
5. Protoplast reagents [26]
  i) Cellulase Onozuka R10 (RPI #: C32200)
  ii) Macerozyme R10 (RPI #: M22010)
  iii) D-Mannitol (PhytoTech Labs#:M562)
  iv) MES (PhytoTech Labs#: M825)
  v) KCl
  vi) CACl_2_*2H_2_O
  vii) BSA
  viii) MgCl_2_*6H_2_O
  ix) NaCl.

### Recipes

1. MS Media (1L)
  - 4.3 g MS salts
  - 1000 µL MS vitamins
  - 20 g Sucrose
  - 400 mg MgSO_4_*7H_2_O
  - Adjust pH to ~ 5.7 adding KOH (HCl can be added if overshot)
  - 3 g Phytagel
  - Autoclave set at 120 °C with 60-min timer
2. Callus induction media (1L) [23]
  - 4.3 g MS salts
  - 1000 µL MS vitamins
  - 20 g sucrose
  - Adjust pH to 5.7 adding KOH (HCl can be added if overshot)
  - 8 g BactoAgar
  - Autoclave set at 120 °C with 60-min timer
  - Add 100 µL 1 mg/mL 2,4-D after autoclave
  - Add 100 µL1 mg/mL 6-BA after autoclave.
3. Protoplast enzyme mix (50 mL) [26]
  - 0.22 g MES
  - Adjust pH to 5.7 adding KOH
  - 0.75 g Cellulase R10
  - 0.2 g Macerozyme R10
  - 3.65 g Mannitol
  - 0.075 g KCl
  - Incubate at 55 °C in 50 mL falcon tube for 45-min
  - Allow to cool to near room temperature
  - Add 0.07 g CaCl_2_*2H_2_0 after incubation
  - Add 0.05 g BSA after incubation
  - Filter entire mixture through a 0.22 µM filter in sterile hood
4. W5 [26]
5. MMG [26].

### Procedures

#### Plant materials development protocols

1. Sterilize and stratify Arabidopsis seeds in microcentrifuge tube.
  - Add 70% ethanol and rotate/resuspend for 5 min.
  - Briefly centrifuge and remove ethanol supernatant
  - Add 1:1 bleach:water (3 or 4% sodium hypochlorite) solution to microcentrifuge tube
  - Rotate/ resuspend for 10 min, but not exceeding 20 min
  - Briefly centrifuge, remove bleach supernatant in sterile hood
  - Add sterile ddH_2_O, close lid and resuspend, briefly centrifuge, remove supernatant.
  - Repeat the ddH_2_O rinse process 5–7 times while leaving the water from the last rinse inside the tube for stratification.
  - Stratify the microcentrifuge in the dark at 4 °C for 4–7 days. Do not go beyond 9 days as this may lead to germination in the tube.
2. Planting of sterile seeds
  - Using sterile conditions, use a pipette to place Arabidopsis seeds on a square plate containing MS media. For diploid plants, sow 4 seeds per grided square.
  - Place into growth chamber for 16/8 h light/dark cycles at 23–24 °C.
3. Transfer to soil
  This method is used for diploid plants that will be crossed.
  - For planting in soil, remove the seedlings from media after ~ 7 days in the growth chamber by gently using a pair of forceps. Be sure to remove the seedlings gently to avoid root damage.
  - Use the forceps to make a small hole in the soil before grabbing the seedling. Place seedling in hole and gently cover roots with soil. *Note—soil should be moist enough to be able to hold itself together but not dripping with water
  - Use a squeeze bottle with a directed stream to dampen the soil and to spray over the covered root section. Avoid spraying the shoot tissue directly.
4. Make crosses for diploid GFP/RFP F1
  *Note—in our tests we only used GFP as a female, we do not know if it makes a difference, however.
  - For the female plant, take unopen flower bud and use fine-tipped forceps and magnifying glasses if needed to gently remove the sepals and petals to expose the pistil and stamen. Gently remove the entire stamen without harming the pistil. *Note—if any anther is left then cross contamination can occur.
  - Tie a piece of fluorescent sewing thread around the emasculated flower bud. Clean the forceps with 70% ethanol.
  - Use the cleaned forceps to remove an open flower from the male donor and rub the anthers on the pistil of the freshly emasculated flower.
  - Be sure to clear the area of potential contaminating anthers/flowers as the emasculated flowers are more susceptible to cross-pollination.
  - Collect the fertile siliques when brown. *Note—Do not let siliques over dry as cracking will occur leading to loss of seeds.
  - Collect F1 seeds, dry at 37 °C for 12-24 h, and store at 4 °C in a dry tube.
5. Plant F1 seeds from the two fluorescent crosses and controls as described in steps 1 and 2.
  - Let seedlings grow in square plate for 20–23 days.
  - Genotype GFP/RFP F1 plants if desired, fluorescent analysis is sufficient.
6. Callus Induction
  - Cut pieces from sterile leaves under sterile conditions with forceps and a sterile razor blade. Simply tearing leaf pieces off with the forceps will also work.
  - Place leaf strips on callus induction media [23]
  - Parafilm plate and place in the dark at 23 °C.
7. Protoplasting
  - After 1–2 months there should be enough calli for protoplasting. Note—needed amount is based on each experiment. Be sure to leave enough for subculturing if culture will be used for subsequent assays.
  - Add 8–10 mL of sterile enzyme solution to a petri dish.
  - Add whitish callus pieces to the petri dish containing the enzyme solution (Amount depends on number of cells needed). *Healthier callus will yield more protoplasts*.
  - Use a sterile razor blade to chop the callus pieces into smaller pieces while in the enzyme solution.
  - Incubate callus tissue in enzyme mix for approximately 3 h in complete darkness under sterile conditions.
  - After this point, cleanliness is required but not sterility.
  - Swirl the enzyme solution and tissue rapidly to further release protoplasts.
  - Add 5 mL of W5 solution to the enzyme and callus containing petri dish1 mL at a time using a circular motion around the petri dish to release the liquid with a pipette.
  - Swirl the enzyme solution and tissue rapidly to further release protoplasts.
  - Use a cut pipette tip to pass solution from petri dish through a 40 µM filter into a 50 mL Falcon tube.
  - Repeat steps h through j
  - Centrifuge 100*g* × 5 min with minimal/medium acceleration and brake.
  - Remove supernatant
  - Add 5 mL W5 to the tube and resuspend the protoplasts gently.
  - Transfer mixture with cut pipette tip to a round bottom, 15 mL culture tube. This is recommended but not critical.
  - Centrifuge 100*g* × 2 min with medium acceleration and brake.
  - Remove supernatant
  - Add MMG to desired concentration of cells.
8. Subculturing callus
  - In sterile conditions, use a sterile set of forceps to remove whitish pieces of callus while leaving leaf fragments behind. Transfer whitish pieces to a fresh petri dish containing callus induction medium. This should be done every 2 to 4 weeks. Callus tissue beyond this point can be subcultured but it is not ideal.
9. Flow cytometry
  - Send protoplasts in MMG solution to be run through a flow cytometer.
  - The instrument sheath fluid is 0.2 µm-filtered 1X PBS.
10. Data analysis
11. As there is proprietary software to work with flow cytometry data, there may be better options to analyze reads, but there are open-source options available, one of which is outlined below.
12. Flow cytometry data comes in .fcs files and cannot be read without special software. To overcome this issue, we used a python package called Flowcal [3] and Pandas [22] to take the raw data files and convert them to .csv files with column names as fluorescent channels for further analysis. The code to write the.csv files is contained in the Jupyter Notebook file found in the Github repository listed below.

The .csv files were then read and further analyzed on R to determine appropriate thresholds for induction tests. Filtering was used to determine maximum and minimum values for various fluorescent channels, thus defining cell populations. The thresholds that we used for analysis are found in Table 2. Scripts for graphing flow cytometry data with ggplot2 version 3.3.6 [24] are provided in the Github repository. ggpubr version 0.4.0 [10] was used to combine multiple plots into a single figure. Generic scripts where variable name can be plugged in are provided in the Github repository listed below for both file conversion and analysis.

#### Optional haploid development

1. To develop haploids, a cross between CS66982 and the two fluorescent diploid lines will need to take place. Since we used the fluorescent diploid lines as the male, we want many flowers to be available for crossing. Plant the male donor 1 to 2 weeks before the CS66982 female to ensure enough flowers.
  - Make crosses for haploids [20].
    i. By using CS66982 as the female, use forceps to gently grab an open Arabidopsis flower from an RFP or GFP plant. Gently rub the anthers of the open flower on the open flower of CS66982 plant. Since CS66982 is mostly male sterile, emasculation is not necessary. This crossing can be made up to 2 weeks after the first flowering of the CS66982. *Note—this can be extended by clipping the bolts and using flowers from the secondary shoots.
    ii. After a cross is made, elongating siliques can be seen approximately 3 to 4 days after making the cross.
    iii. Collect the fertile siliques for haploids when dry. *Note—Do not let siliques over dry as cracking will occur leading to loss of seeds.
    iv. Collect F1s, dry in 37 °C for 12–24 h, and store at 4 °C in a dry tube.
2. Plant F1’s from the CS66982 and GFP or RFP cross as described above.
3. For haploid plants, transfer to MS Magenta boxes.
  - Under sterile conditions, use a set of forceps to gently remove the seedlings from the media after 7 to 9 days in the growth chamber –*small plants should be allowed to grow until roots can be placed under media after transferring*.
  - Use the forceps to make a small hole in the media of the Magenta box containing MS media before grabbing the seedling. Place seedling in the hole and gently cover roots with media. *Note—Root damage can make differentiation of haploids, diploids, and aneuploids nearly impossible by phenotyping. Be sure to be gentle.
  - Place Magenta boxes in growth chamber under the same conditions as described above.
    i. Do the same with a diploid control in order to phenotype haploids.
4. If haploid plants are used, phenotype to select true haploids.
  - Follow steps from Ravi and Chan [20].

### Supplementary Information

**Additional file 1.**Histograms corresponding to the various spike-ins and cell classifications for each data analysis technique for FITCA and PECF594A.

## Data Availability

The datasets used and/or analyzed during the current study are available from the corresponding author on reasonable request. Blank scripts to ensure that anyone with even a limited bioinformatics background can convert.fcs files to.csv files (Jupyter notebook) and analyze them using RStudio can also be found at the link. https://github.com/Lubberstedt/High-throughput-meiosis-induction-detection_methods. RFP and GFP lines have been made available at ABRC (https://abrc.osu.edu/; Stock numbers CS73495 and CS73496, respectively).
