# Supplementary material for: Assessing data analysis techniques in a high-throughput meiosis-like induction detection system
Source: Plant Methods. 2024 Jan 12;20:7. doi: 10.1186/s13007-023-01132-9 (PMC10785433; doi:10.1186/s13007-023-01132-9)

FITCA: RFP and GFP w/o Quality

Key:
GGR = GFP and GxR classified cells collated together

RFP = RFP classified cells

WT = non-fluorescing classified cells

Percentage = RFP spiked-in

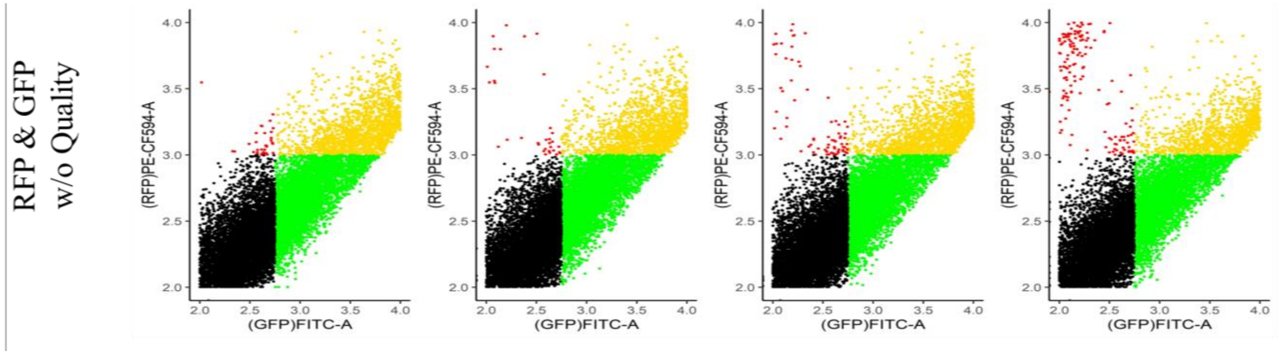

GGR | RFP | WT

0%

0.28%

1.18%

3.89%

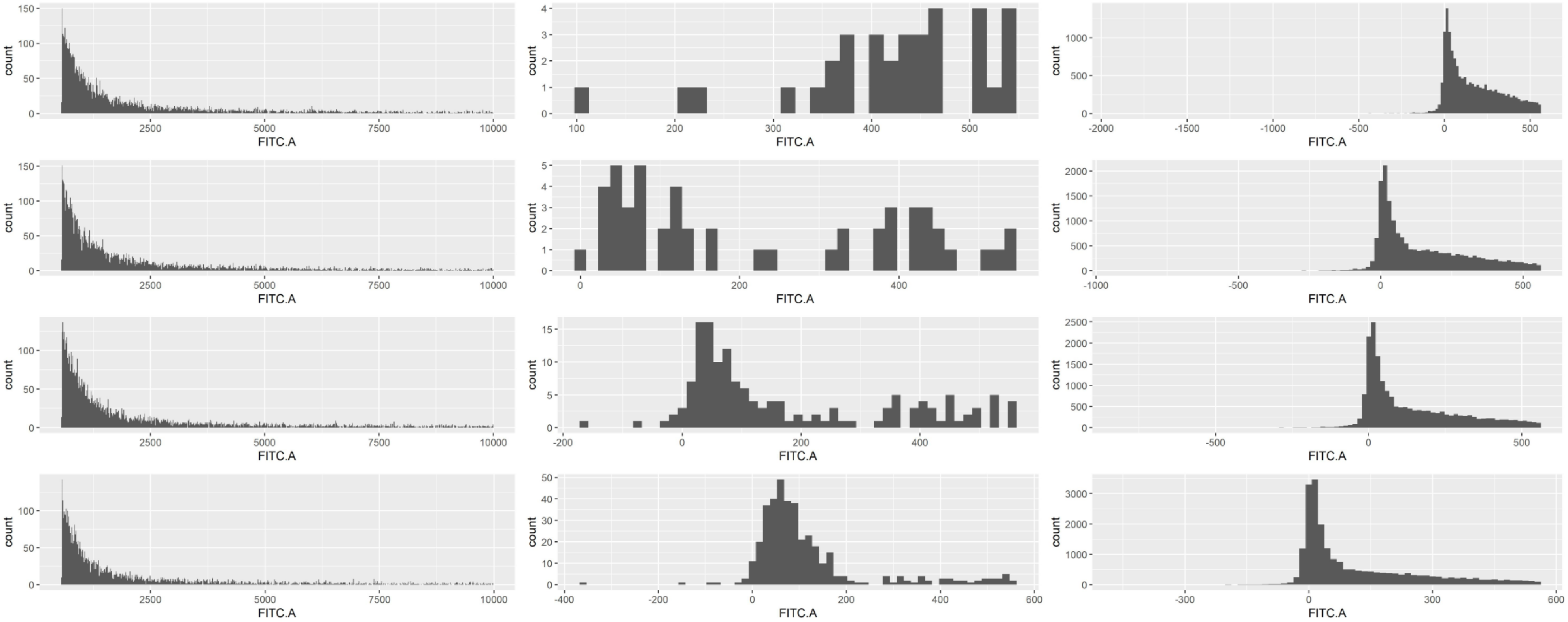

PECF594A: RFP and GFP w/o Quality

Key:
GGR = GFP and GxR classified cells collated together

RFP = RFP classified cells

WT = non-fluorescing classified cells

Percentage = RFP spiked-in

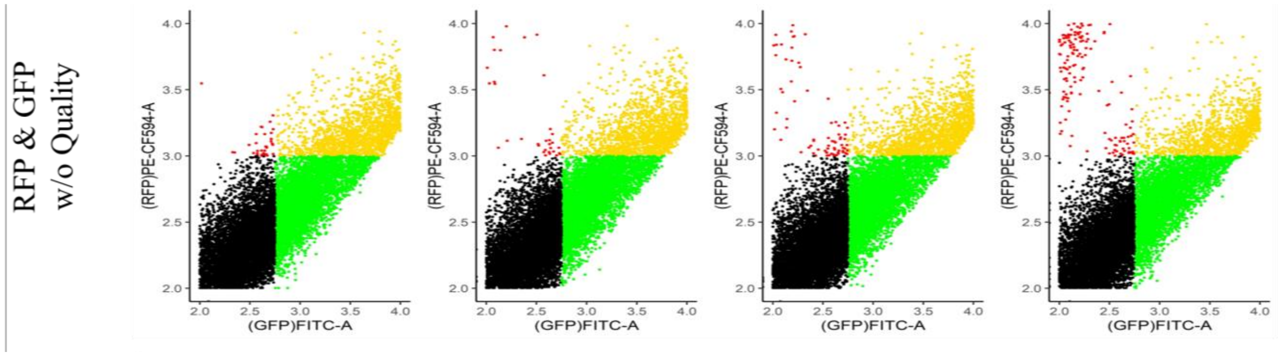

GGR | RFP | WT

0%

0.28%

1.18%

3.89%

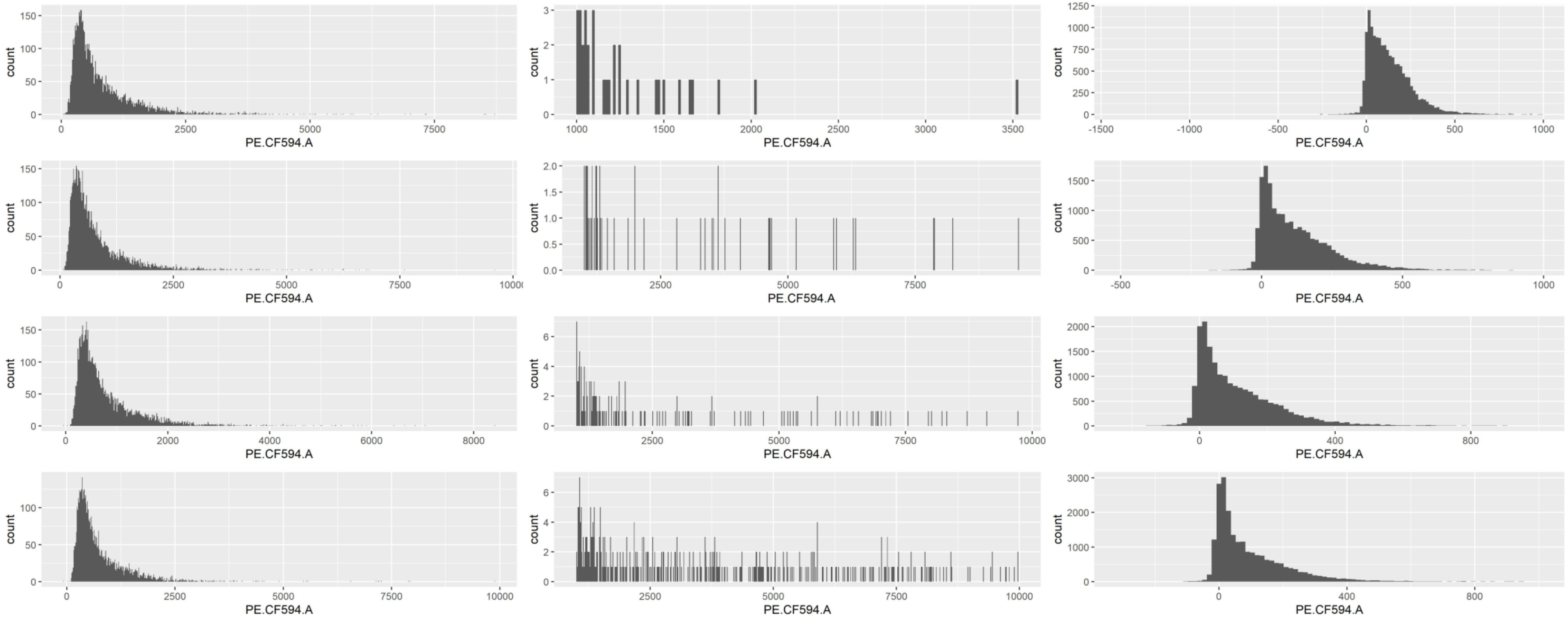

FITCA : RFP and GFP w/Quality

Key:
GGR = GFP and GxR classified cells collated together

RFP = RFP classified cells

WT = non-fluorescing classified cells

Percentage = RFP spiked-in

RFP & GFP w/ Quality

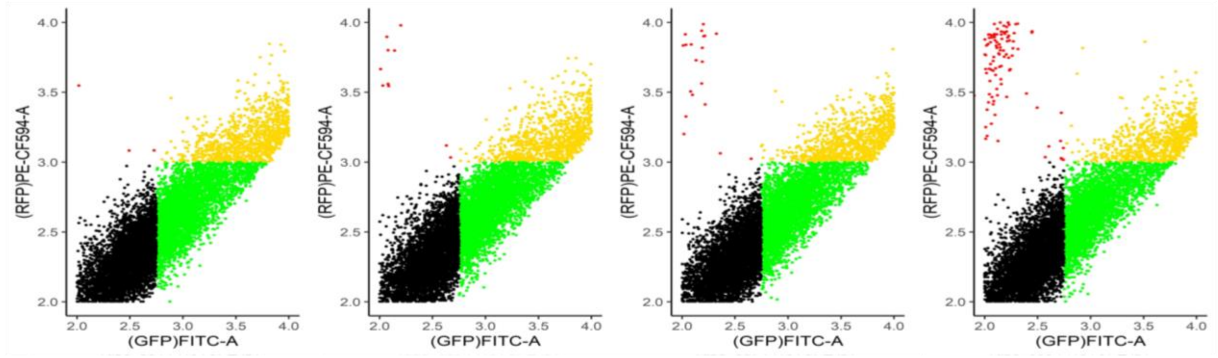

GGR | RFP | WT

0%

0.28%

1.18%

3.89%

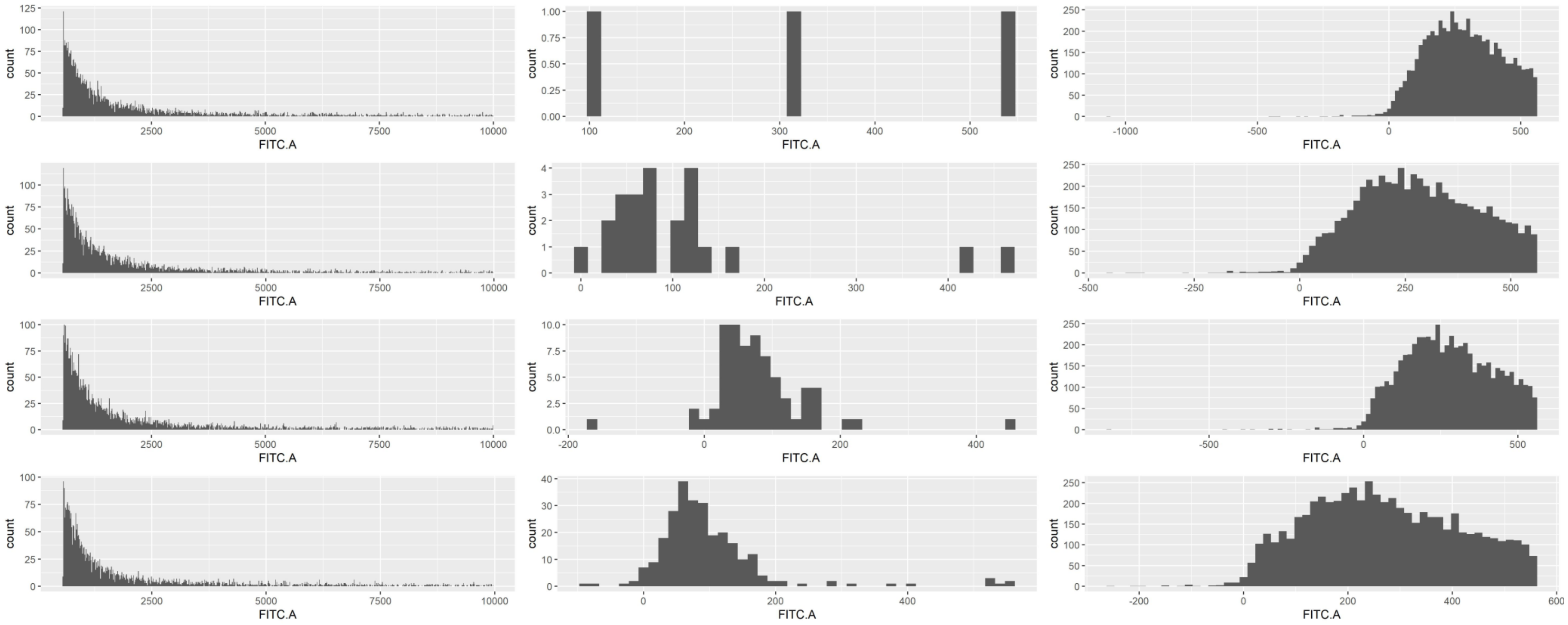

PECF594A : RFP and GFP w/Quality

Key:
GGR = GFP and GxR classified cells collated together

RFP = RFP classified cells

WT = non-fluorescing classified cells

Percentage = RFP spiked-in

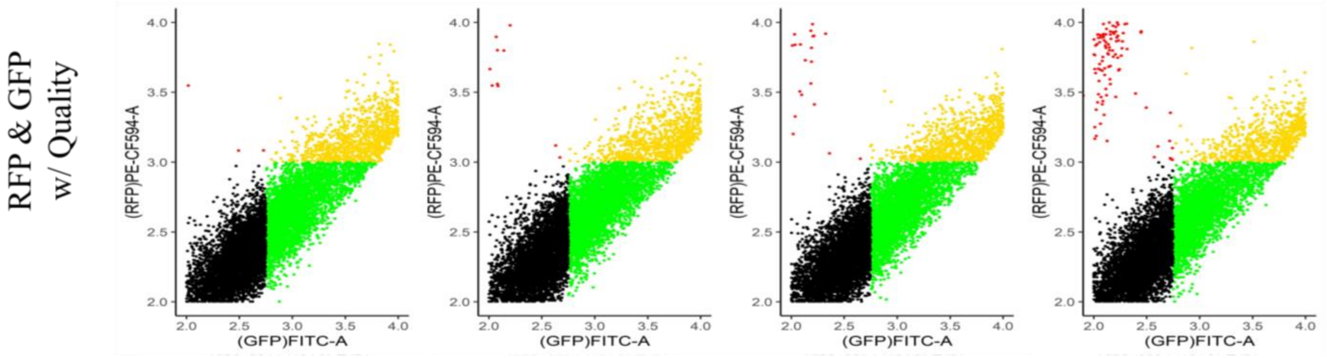

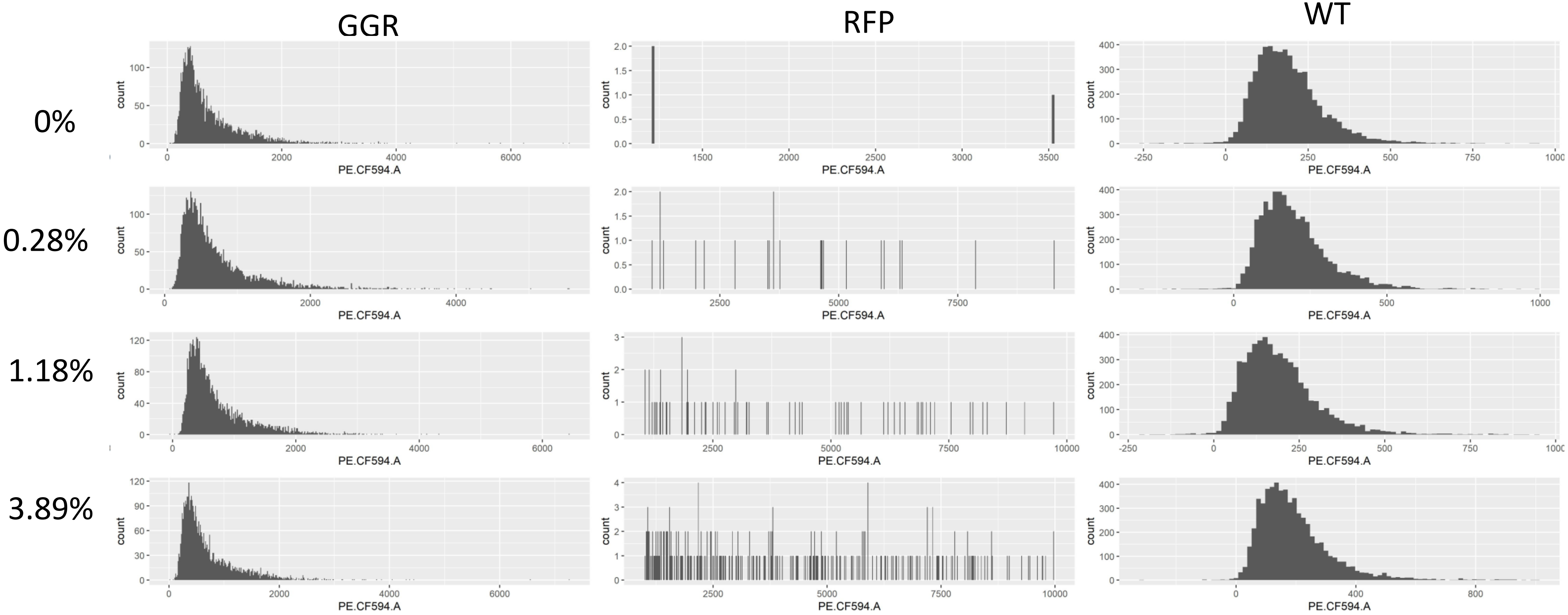

FITCA : Polygonal Gating

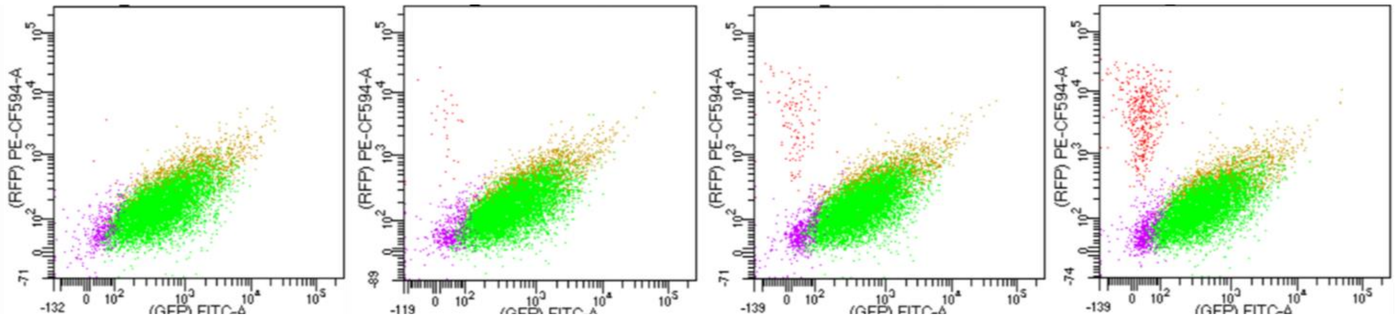

Key:
GGR = GFP and GxR classified cells collated together

RFP = RFP classified cells

WT = non-fluorescing classified cells

Percentage = RFP spiked-in

GGR | RFP | WT

0%

0.28%

1.18%

3.89%

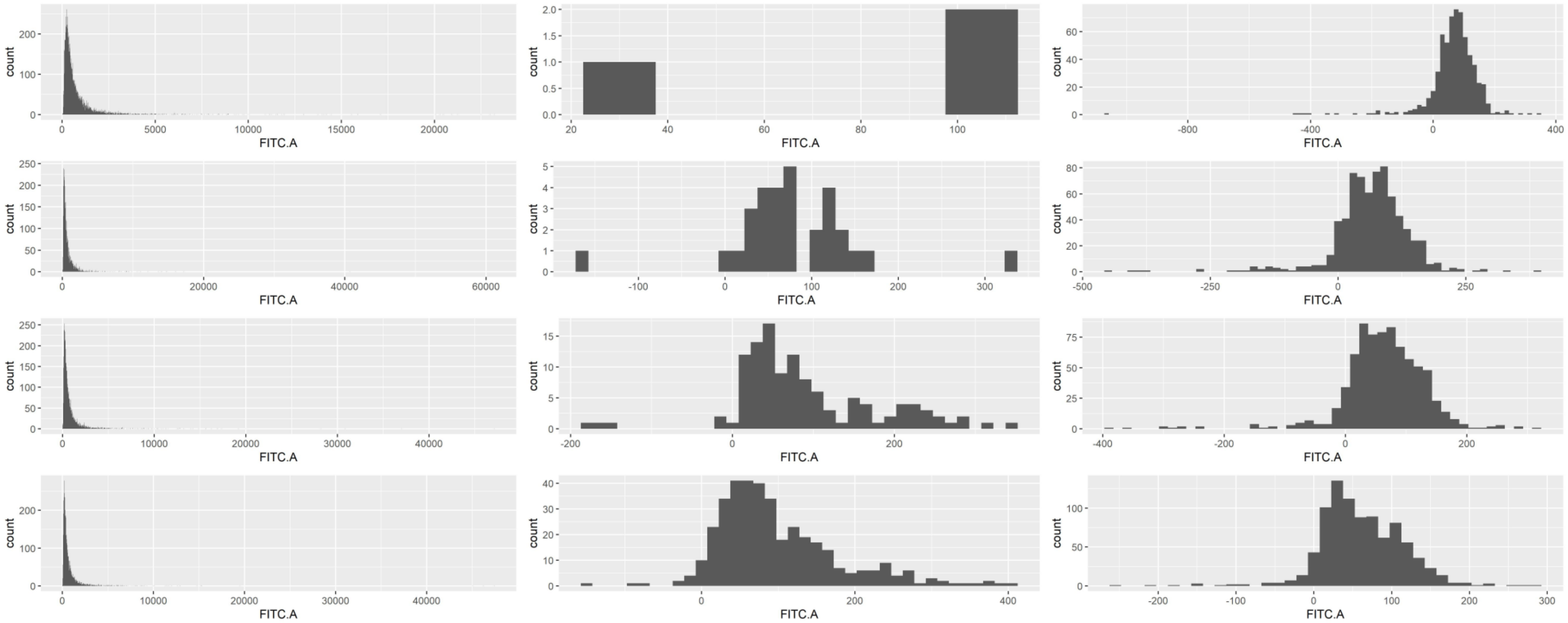

PECF594A : Polygonal Gating

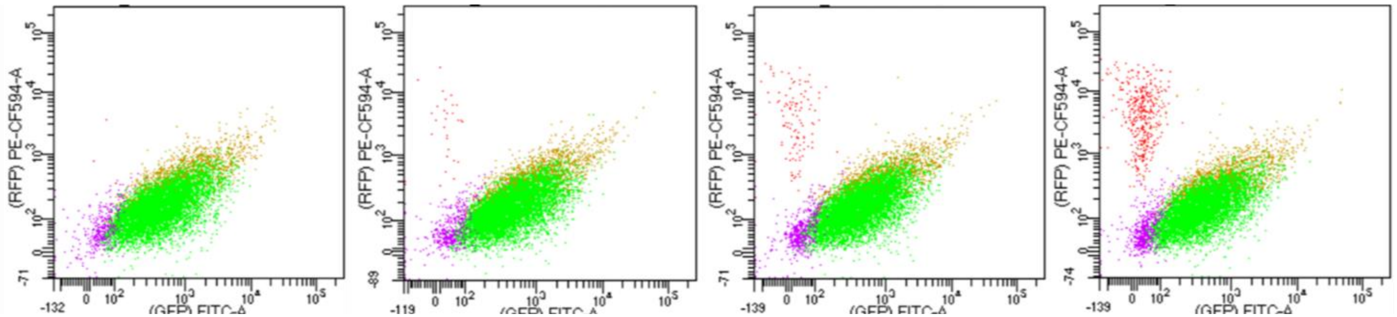

Key:

GGR = GFP and GxR classified cells collated together

RFP = RFP classified cells

WT = non-fluorescing classified cells

Percentage = RFP spiked-in

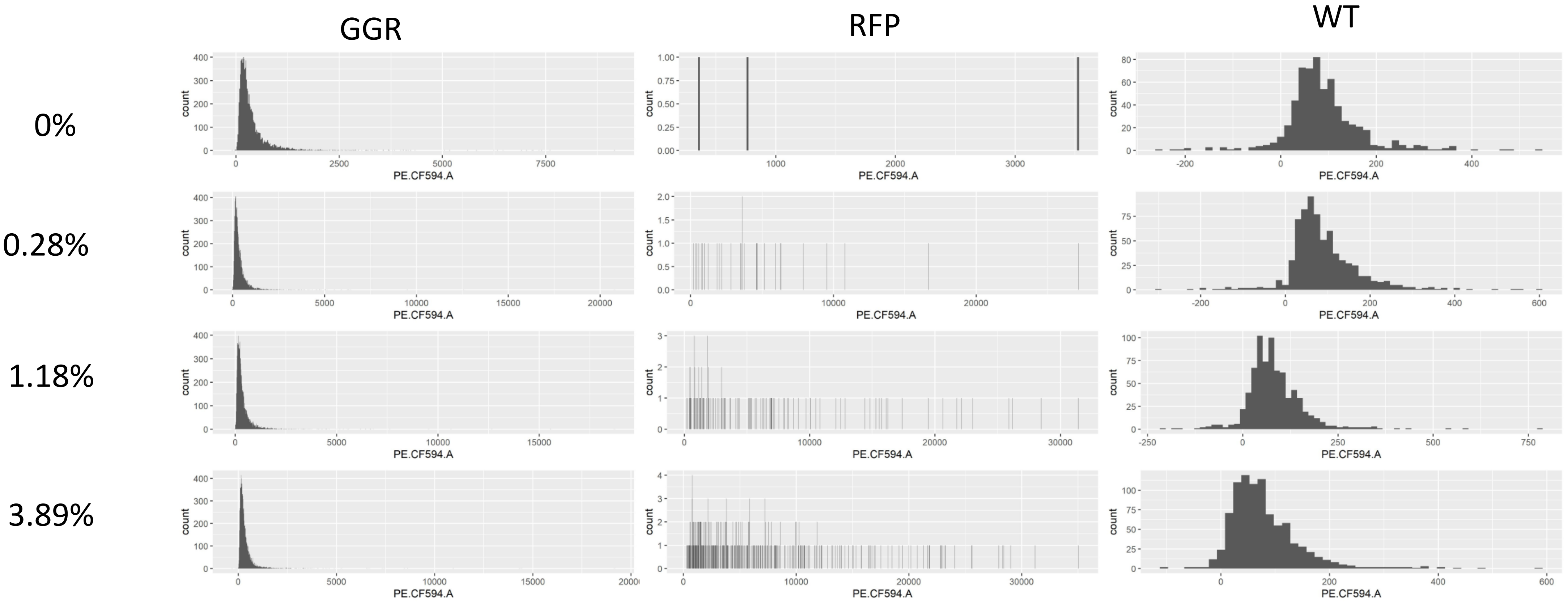

Supplement: Supplementary file 1 — Additional file 1.Histograms corresponding to the various spike-ins and cell classifications for each data analysis technique for FITCA and PECF594A. [file 13007_2023_1132_MOESM1_ESM.pdf]
